# Cross-Cultural Patient Counseling and Communication in the Integrative Medicine Setting: Respecting the Patient's Health Belief Model of Care

**DOI:** 10.1007/s11920-024-01515-2

**Published:** 2024-06-17

**Authors:** Eran Ben-Arye, Gabriel Lopez, Maryam Rassouli, Miriam Ortiz, Holger Cramer, Noah Samuels

**Affiliations:** 1 Rappaport Faculty of Medicine, Technion-Israel Institute of Technology, Haifa, Israel; 2 Integrative Oncology Program, The Oncology Service, Lin, Zebulun, and Carmel Medical Centers, Clalit Health Services, Haifa, Israel; 3 Department of Palliative, Rehabilitation and Integrative Medicine, University of Texas, MD Anderson Cancer Center, Houston, TX USA; 4 Cancer Research Center, Shahid Beheshti University of Medical Sciences, Teheran, Iran; 5 Charité - Universitätsmedizin Berlin, Corporate Member of Freie Universität Berlin and Humboldt-Universität zu Berlin, Institute of Social Medicine, Epidemiology and Health Economics, Berlin, Germany; 6 Institute of General Practice and Interprofessional Care, University Hospital Tübingen, Tübingen, Germany; 7 Robert Bosch Center for Integrative Medicine and Health, Bosch Health Campus, Stuttgart, Germany; 8 Center for Integrative Complementary Medicine, Shaarei Zedek Medical Center, Faculty of Medicine, Hebrew University of Jerusalem, Jerusalem, Israel

**Keywords:** Cross-cultural medicine, Mental health, Integrative medicine, Integrative oncology, Psycho-oncology, Doctor-patient communication, Health-belief model

## Abstract

**Purpose of Review:**

Communicating effectively with patients having a traditional, alternative or complementary medicine-related health-belief model is challenging in today’s cross-cultural society. This narrative review explores the integrative medicine setting of care, focusing on insights from the integrative oncology daily practice, while addressing the relevance to the mental health setting. The way in which healthcare providers can enhance cultural-sensitive communication with patients and informal caregivers; recognize and respect health-beliefs to bridge cultural gaps; and generate an open, non-judgmental and mindful dialogue are discussed.

**Recent Findings:**

Identifying cross-cultural barriers to healthcare provider-patient communication is important in order to address the potential for conflict between conventional and “alternative” health beliefs; difficulties in creating a shared-decision making process; disagreement on therapeutic goals and treatment plan; and finally, the potential for non-compliance or non-adherence to the conventional oncology treatment.

**Summary:**

Acquiring intercultural competencies is needed at all stages of medical education, and should be implemented in medical and nursing curricula, as well as during specialization and sub-specialization. As with patient-centered paradigms of care, integrative medicine entails a dual patient-centered and sensitive-cultural approach, based on a comprehensive bio-psycho-social-spiritual model of care.

## Introduction

The changing demographic of Western countries, with a significant increase in recent years in their refugee populations, has presented a number of cultural-related challenges to the medical establishment [1]. These reflect the traditional cultural composition of varied Western societies, which are undergoing a transformation in its encounter with the mosaic of first- and second-generation immigrants from a variety of cultures and health belief models. Interculturality, the egalitarian interaction between cultures with varied ethnic makeup, religion, language and nationality, is becoming increasingly challenging to healthcare provider (HCP)-patient interactions (see Table 1). Communicating with a person seeking medical care requires recognizing and respecting the patient’s attributes, behaviors and health-beliefs, which may oppose those of the HCP. This is also true for HCPs providing complementary and integrative medicine (CIM) to refugees and other minority groups, whose practices and beliefs may also contrast with those of Western-based integrative medicine [2]. Despite the limited research on this subject, a cross-sectional survey of German immigrants from Turkey and the former Soviet Union found significant use of home-made remedies, as well as a sense of “feeling misunderstood” vis-a-vis cultural needs provided by German HCPs [3].

**Table 1:** Potential risks of not adapting a cultural-sensitive TCAM-related consultation

**Key themes**	**Clinical setting**	**Specific aspects**	**Evidence type** (see references)
**Non-disclosure of TCAM**	**Oncology setting**	The lack of adequate discussion about TCAM use can increase the risk of negative interactions with conventional cancer treatments, and a missed opportunity for HCPs and patients to engage in vital exchange of information.	Systematic review [79]
**Proactive and non-judgmental questioning about TCAM use**	**Diabetes**	Enhanced communication has been reported among HCPs who openly and pro-actively asking low income and racially diverse patients about TCAM use. Lack of pro-active questioning may impair shared decision-making.	Patient survey; Qualitative research [80]
**Risk of not respecting the patient’s beliefs**	**Oncology**	HCPs are obliged to discuss relevant concerns while respecting the patient’s health beliefs, as well as cultural and linguistic diversity.	Systematic review [81]
**Lack of HCP training in TCAM**	**Pediatric oncology**	A lack of national and regional standardized training programs for supportive care and TCAM in pediatric oncology may be associated with direct (treatment-related) and indirect (e.g., lack of professionalism of TCAM providers, avoidance of conventional care) risks.	Systematic review [82]
**Lack of addressing TCAM-related safety issues**	**Oncology**	Based on 720 questionnaires obtained in five German oncological practices, potential interactions with anti-cancer drugs were identified in 39% of vitamin and 17% of herbal supplements. Lack of structured communication and safety evaluation may increase the risk for a negative interaction.	Systematic evaluation of potential interactions [83]
**Risk of non-adherence to conventional care**	**Stroke care**	The most common reason for inadequate stroke therapy and higher dependence on TCAM was the patients' lack of knowledge and the complexity of the conventional medication regimen.	Meta-analysis and Systematic Review [84]
**Unmet needs of informal caregivers**	**Pediatric oncology**	Parents demand respect for the family's autonomy when choosing TCAM for their children.	Integrative medicine systematic review [85]

*TCAM* Traditional, complementary, and alternative medicine

This narrative review explores the challenges facing HCP-patient communication in clinical settings such as integrative oncology (IO), psycho-oncology, and palliative care, in which evidence-based complementary practices, including mind-body interventions, are part of conventional care and clinical practice guidelines [4, 5]. The IO setting will be used to address the study’s primary objective of presenting a cross-cultural approach to facilitating HCP-patient communication on the use of traditional, complementary and/or alternative medicine (TCAM). This, while acknowledging the relevance of discussed themes in the integrative mental health therapeutic setting. The benefits of a non-judgmental, culture-sensitive dialogue will be highlighted, as will the risks of not doing so, especially in minority groups where TCAM is associated with the patients’ collectivistic identity and affinity to traditional medicine [6]. Here, inter-cultural communication can be further polarized from the conflict between conventional and “alternative” approaches to medical care, the latter prevalent among patients with a high affinity to traditional medical practices which often contradict, even negating evidence-based conventional oncology care.

## Methods

In order to approach this multidisciplinary and multi-cultural subject, a team of six co-authors from Germany, Israel, Iran and the United States was selected (four physicians, one nurse, one psychologist and one anthropologist), all with experience in cross-cultural communication. Databases were searched for explanatory (i.e., randomized, controlled trials), pragmatic research, qualitative research, meta-analysis and systematic reviews using MEDLINE/Pubmed; Cochrane Database of Systematic Reviews; and the Memorial Sloan-Kettering Integrative Medicine Service Database. Key words searched included the following: alternative/complementary/traditional/integrative medicine; doctor-patient communication, oncology, psycho-oncology, palliative care; supportive care; disclosure, refugees, minorities, underprivileged, diversity, equity and inclusion. The scientific literature was searched from 2000, with the authors providing personal, scientific, clinical and medical education-related experiences. The findings and varying perspectives were then summarized, with the meaningful themes elicited discussed.

### Challenges Facing Cross-Cultural Communication

Cross-cultural challenges in healthcare can result from language-related barriers, as well as varying health beliefs and concepts regarding the healing process. These can prevent effective HCP-patient communication (see Table 1), with patients from cultural backgrounds different from their HCPs frequently reporting that their needs are not being met. Access to healthcare services may be limited in vulnerable groups such elderly and oncology patients because of language, cultural, religious, knowledge and financial barriers [7]; and culture-based gender roles can make it difficult for female patients to seek medical care [8]. In an Iranian study, Khoshnazar et al. found an expressed desire among female patients with breast cancer for therapeutic communication focused on empathy, and not merely “sharing knowledge” [9]. Other research has shown that Muslim patients perceive their physical and spiritual suffering as a process of atoning for their sins, and thus an opportunity for religious purification [10]. This model may challenge the European perception of pain, making cross-cultural HCP-patient communication even more difficult [11].

HCP communication with minority groups can enhance cross-cultural skills, adopting a patient-centered holistic approach. This can include the “bio-psychosocial model”, coined in 1977 by the American psychiatrist George Engel, suggested that Western society’s biomedical model has become “our own culturally specific perspective about disease …. our (own) folk model [12],.” Oncologists Epner and Bailey later posited that the multicultural approach to cultural competencies can lead to stereotypical thinking, in which culture is “a very elusive and nebulous concept, like art [13].” Degrie et al.’s systematic review of qualitative research of the intercultural care encounter concluded that difficulties in HCP-patient communication could arise from differences in perceptions of health and illness; religion; the primary caregiver’s role, and language-related barriers [14].

This cross-cultural approach was further discussed by Kleinman et al. regarding cultural challenges via a transactional model based on medical and cultural anthropology, with patients and HCPs learning about each other’s explanatory models for disease and illness (“the human experience of sickness”) during the clinical encounter [15].

### Challenges Facing the “Alternative Medicine” Conversation

Establishing a dialogue between oncology HCPs and patients with a TCAM health-belief model may encounter a number of inter-cultural challenges. This may require addressing the potential stigma among HCPs towards immigrants and other minorities using traditional medicine. This has been examined in a systematic and narrative synthesis of qualitative studies which ask the question of how chronic conditions are understood, experienced and managed within African communities in Europe, North America and Australia [16]. Reese et al. from the University of Montana in the U.S. conducted a qualitative study among members of a state-recognized Gulf Coast tribe [17]. Emergent themes included a preference for and use of traditional medicine, and for a holistic approach to health. At the same time, they identified negative HCP-related interpersonal interactions, which were associated with a reluctance to seek medical care, and a resistance to Western healthcare systems.

In a Swiss study by Walthert et al., self-perceived discrimination was reported among 36% of asylum seekers, more so among those using TCAM modalities which often included the use of traditional herbal medicine [18]. HCP-related stigma, prejudice, and bias toward TCAM use by patients can be seen in both inpatient and outpatient settings of care. In an Israeli study, the non-referral by HCPs to an integrative oncology consultation, provided without charge, was associated with patients' ethno-national origin and immigration status (primary language, Arabic and Russian), as well as male gender and older age [19]. Discrepancies in the provision of healthare may be also be seen in situations in which the health-belief models of the patient vary greatly from those of the HCP. This has been seen with respect to the needs of spiritual care among parents caring for children with a life-threatening illness at home [20].

At least half of patients using herbal medicine and other TCAM modalities do not disclose this to their HCPs, especially oncologists and pharmacists [21]. Data on non-disclosure of TCAM use among psychiatry patients is limited, despite the high prevalence of this practice (43%) among patients who met DSM-IV criteria for Generalized Anxiety Disorder (GAD), Panic Disorder, Social Anxiety Disorder, or Post-Traumatic Stress Disorder [22]. In the pediatric setting, less than one-third of parents to children with attention deficient/hyperactive disorder were found to disclose their children's use of TCAM to their medical doctors [23].

Non-disclosure, ranging from 20 to 77%, has been attributed to an anticipated negative or dismissive response; assumption that the HCP lacks knowledge on the subject; or the HCP not raising the subject, due to a lack of awareness, training, time constraints or simply forgetting to ask [24]. Kelak et al. found that HCPs who take the time to listen attentively and respectfully are more likely to have patients disclose TCAM use [25].

Culture-related barriers to patient use of freely available CIM services by Arabic-speakers in Israel have been attributed to two separate paradigms of folk-traditional medicine, of either consulting with “healers” as opposed to the integrative approach based on the bio-medical model of care [26]. Patient disclosure of TCAM use (34% in Israeli hospitals vs. 70% in primary care settings) and the HCPs’ inclination to initiate a discussion about these practices may depend on how each contextualizes non-conventional medical practices [27]. If seen as ‘alternative’ and contrary to mainstream medicine, such a discussion might be perceived by both parties as irrelevant and thus not raised in the discussion of treatment options. However, if perceived within a more ‘integrative’ framework, it is more likely that TCAM use will be a topic for discussion [28]. The transition from a “traditional-alternative” to a “traditional-integrative” approach to care is being promoted by the World Health Organization’s Traditional Medicine Strategy (2014–2023), which has called for the integration of traditional medicine in national healthcare systems [29].

In their systematic review, Tangkiatkumjai et al. suggested that TCAM use can be accompanied by an expectation of benefit; perception of safety; and dissatisfaction with conventional medicine [30]. Patients and HCPs who see TCAM as ‘natural’ and without risk may be unaware of potentially harmful effects, such as interactions between herbal products and cancer drugs. A regional survey of HCPs in 16 Middle-Eastern countries identified potential risks such as altered pharmacodynamics; directly toxic effects; and increased in vitro response of cancer cells to chemotherapy drugs [31].

Unrealistic expectations from TCAM use, such as “alternative” treatments that can “cure” cancer, contrast with those of the HCP who sees evidence-based TCAM as addressing quality of life-related concerns only [32]. In the multi-cultural context of care, expectations regarding “cures” by “wonder drugs” or other TCAM modalities may be reinforced by the patient’s informal caregiver [33]. Such expectations may reflect spiritual and religious practices; health-related traditions; and activities such as praying, or reciting the Qur'an (or other sacred books in other religions), all of which may contradict the conventional paradigm of medical care [34].

### The Integrative Approach: Bridging the Cross-Cultural Communication Gap

Over the past two decades, the concept “complementary medicine” has been evolving into the “integrative medicine” setting, with evidence-based TCAM modalities incorporated within the core of mainstream medicine. This has been most prominent in integrative oncology (IO) settings in mainstream oncology and palliative care services across the globe.

Most IO care starts from the initial “breaking of bad news”, continuing throughout the stages of the cancer journey, including neo-adjuvant, peri-operative, adjuvant therapies and survivorship, and end-of-life care. Most IO programs involve a multi-disciplinary team of physicians, nurses and other HCPs with dual training in conventional supportive care and TCAM-based modalities [35]. Therapies such as acupuncture, touch and mind-body modalities are frequently combined, in accordance with evidence- and guideline-based medical practice [36, 37]. The goals of IO programs focus on quality of life-related concerns, supported by clinical research for oncology treatment-related toxicities (e.g., chemotherapy-induced peripheral neuropathy [38]) and peri-operative concerns (e.g., preoperative anxiety and postoperative pain [39]). These programs have been shown to increase patient adherence to oncology treatment regimens**,** within a safe and effective environment [40].

Many of today’s European cancer centers provide IO care to patients (e.g., Baden-Württemberg, Germany; Tuscany, Italy). In the U.S, IO services can be found working in collaboration with psycho-oncology and palliative care teams in leading oncology centers including at the Memorial Sloan-Kettering Cancer Center in New York; Dana-Farber Cancer Institute in Boston; and at the MD Anderson Cancer Center in Texas [41–43]. For over two decades the Society for Integrative Oncology in the United States, with ambassadorship programs in Europe and the Middle East, has been promoting international research, clinical, and medical education projects based on a patient-centered bio-psycho-social-spiritual model of care, with a commitment to diversity, equity, and inclusion (DEI) of care. While IO is seen as a promoter of a culturally-sensitive approach to patient care [44], in countries such as Israel a number of DEI-related challenges have been identified, attributed to either HCP- or accessibility-related barriers, with non-Hebrew-speaking patients less likely be referred to a freely-provided IO consultation [45].

Patient trust in their HCP has been shown to increase when asked directly about TCAM use, providing a non-biased risk–benefit evaluation with a greater chance for disclosure of these practices [12]. It is important to focus on past experience with TCAM, expectations from the IO consultation, and involvement in co-designing the IO treatment plan [46]. Information provided during IO consultations can facilitate a dialogue in which integrative physicians can address the patient’s culture and health belief system, helping make an informed decision regarding TCAM options [47]. Dually-trained integrative HCPs who understand both conventional and TCAM “languages” of care can facilitate this HCP-patient dialogue, serving as “gatekeepers” to ensure an effective and safe therapeutic environment [21].

### TCAM-Related Cross-Cultural Challenges Specific to Mental Health

The prevalence of TCAM use in mental health is considered an important subject for research in various settings of care. The World Mental Health Surveys, conducted across 25 countries, found that 3.6% of the population with a diagnosis of a mental health disorder had reportedly been using TCAM over the previous 12-month period, more frequently among those from a high-income country when compared to low- and middle-income countries [48]. Higher rates of TCAM use were found among those with more severe illness, with the prevalence of this practice ranging from 14% among those receiving specialist mental health care for severe mood disorders; to 16% among those with severe anxiety disorders and 22.5% with severe behavioral disorders. Higher rates for TCAM use were also found in acute care hospitals (Germany) [49] and health care units treating patients with psychiatric symptoms (Sweden, reported use of 62%) [50]. In the U.S., TCAM use was found to be significantly more prevalent among children with mental health issues when compared to those without (19% vs. 10%), with the majority perceiving TCAM as helpful, natural, and holistic [51].

It is especially important when examining the role of TCAM in mental health to address the stigma which may accompany the diagnosis of a mental illness. In many societies across the globe, the use of TCAM may be a more acceptable option in treating any form of mental illness. For example, a cross-sectional epidemiological survey of mental disorders in China found that very few patients with a one-year diagnosis of a depressive disorder were receiving psychiatric care (2.7%), with many turning instead to traditional Chinese medicine for treatment [52]. Data from the 2016 Singapore Mental Health Study found that Malays reported using primarily TCAM practices to treat mental illness, while among patients from the Indian and Chinese population TCAM use was associated with poorer mental health-related quality of life [53]. In the U.S., race and ethnicity-associated parameters were associated with the use of TCAM among adults with moderate mental distress, with a significantly lower prevalence among the African American population, when compare to Asians and other racial groups [54].

The use of TCAM by patients in the psychiatric setting can present a number of challenges to patient-HCP communication, especially when there is a gap between the patient's health belief models and that of the HCP, as well as their affinity toward traditional medical practices (e.g., herbs, spiritual modalities); the scientific evidence supporting these practices; and the expectations of the patient and their informal caregivers. This potential communication gap may be associated with a reluctance on the part of the patient to disclose their use of TCAM, as shown in an Australian study of adults experiencing anxiety, among which 48% did not disclose TCAM use to their HCPs [55].

Patient-HCP communication-related gaps in the mental health setting may also be related to cross-cultural factors, including an affinity of the patient to traditional medicine modalities such as spiritual practices and herbal medicine. In a cross-cultural study from the Kingdom of Saudi-Arabia, among patients with a psychiatric diagnosis (44% with depression) 82% reported using one or more TCAM modalities during the previous year to address their mental illness, most commonly spiritual therapies such as Quran recitation and mind-body therapies [56]. A similar preference for spirituality and traditional medicine was reported in a study of Cambodian patients with depressive symptoms, who reported consulting with an herbalist and practicing visualization and praying for their wellbeing and health [57]. In a cross-sectional survey conducted in 6 European countries, patients who during their lifetime had sought out psychological help reported consulting with TCAM providers (e.g., herbalists), often with religious figures as well (e.g., ministers, priests, or rabbis) [58].

Patients with mental health-related illness are similar in many ways to the population of patients with cancer. Both groups frequently seek out TCAM practices in a wide range of contexts (traditional, alternative, complementary, and integrative settings of care), together with conventional healthcare. The contextualization of TCAM in both settings as being separate from conventional health care, or in parallel to or within an integrative care setting, all involve a number of participants, including patients and their informal caregivers, HCPs, TCAM practitioners, and others. From the patient's perspective, self-treatment with TCAM modalities (e.g., self-acupressure; meditation; dietary changes; etc.) may be seen as empowering the role they play in their medical treatment, whether it be for mental health or for cancer. Pirotta et al. examined a cohort of Australian patients with depressive symptoms, who were using herbal medicine in what they considered as the main aspect of their treatment [59]. In the UK, Rüdell et al. found that primary care patients with mental health issues were frequently turning to alternative medicine in the form of traditional healers [60]. In Kenya, Musyimi et al. explored the views of traditional health practitioners, highlighting the challenges as well as potential for collaboration with mental health professionals [61]. Finally, in Ghana, Kpobi and Swartz found widely varying concepts regarding TCAM and the potential for collaboration and integration with conventional mental health services [62].

As mentioned, there has been little published on the integration of TCAM in conventional mental health care, including on the perspective of mental health professionals. Liem explored attitudes among clinical psychologists in Indonesia, identifying the potential for including TCAM in the clinical psychology service [63]. Participants recognized TCAM as an essential part of Indonesian culture, and supported the inclusion of TCAM education in psychology education [64]. In a qualitative study among Australian psychologists examining their perceptions regarding TCAM, some were found to be open to the use of some forms of TCAM to enhance patient care, though they were still skeptical vis-à-vis the ethical constraints preventing integration [65]. In Norway, Stub et al. conducted individual and focus group interviews in a “Psychosomatics Clinic” which had introduced craniosacral therapy for patients with complex traumas, including post-traumatic stress disorder [66]. In the end, however, the integrative process in mental health is still far behind what has been taking place in the field of integrative oncology, with the exception of the few mental health initiatives mentioned earlier in the Netherlands, the US, and Israel [67–69]. Further research is thus needed to explore the implementation of integrative models of mental health care, identifying barriers and facilitators to integration, including from cultural-sensitive perspectives.

### Practical Suggestions

Several integrative medicine models have been suggested to facilitate the acquisition and development of cultural-related competencies among oncology HCPs. In their systematic literature review, Brown et al. identified migration, acculturation and expectations as significant challenges to patient care, emphasizing the need for a respectful and empathic attitude toward and awareness of HCP-related cultural barriers [70]. In the multicultural community of Singapore, Tay et al. explored narratives among nurses, emphasizing their central role in bridging communication barriers with hospitalized oncology patients [71]. This role is extremely important for patients and informal caregivers from a culture in which talking about cancer, including prognosis and possible need for end-of-life care is unacceptable due to associated stigma [5].

Purnell et al. described a framework in which oncology HCPs can successfully engage with patients, taking into account a number of contextual factors, including the patient’s society, community and family, as well as their individual role regarding elements contributing to the complexity of what is defined as “culture” [72]. The model described presents 12 cultural domains providing a framework for HCPs, helping them understand their own cultural beliefs, attitudes, values, practices, and behaviors as well. This, with the goal of facilitating the delivery of consciously sensitive and competent healthcare. In this regard, TCAM use in conjunction with conventional oncology care may often require HCPs to communicate from a collectivistic perspective, acknowledging the patient’s affinity, as well as that of their informal caregivers and community, to traditional medicine. This can conflict with the Western-individualistic approach, which emphasizes patient-centered care and patient-tailored treatments. The cultural-sensitive approach needs to be more patient-community centered, tailoring the treatment program with family and community members, in a shared-decision making process. The therapeutic alliance between individualistic and collectivistic perspectives requires an acknowledgement of the patient regarding health beliefs and values.

The LEARN (Listen, Explain, Acknowledge, Recommend, and Negotiate) model, proposed by Berlin and Fowkes as a practical framework for HCPs engaging in cross-cultural conversations, could be extremely helpful for this purpose. This framework suggests *listening* to the patient with sympathy and understanding regarding the patient's perception of the problem, while *explaining* the HCP’s perception and *acknowledging* and discussing the differences and similarities. This is followed by *recommending* treatment and *negotiating* agreement, the latter perceived as the key concept of the LEARN model: “*The final treatment plan should be an amalgamation resulting from a unique partnership in decision making between provider and patient. A patient can truly be involved in the instrumentation of recovery if the therapeutic process fits within the cultural framework of healing and health*” [73].

 In addition to communication skills and awareness, it is also important to acknowledge assumptions and stereotypes among HCPs regarding TCAM and TCAM users [74]. Caspi et al. has suggested that categorizing the many traditional and non-conventional modalities under one definition, that of *complementary and alternative medicine,* generated from the physician's perspective of “numerous stereotypes, prejudices, and misconceptions that may compromise the therapeutic relationship” [75]. Nápoles-Springer et al. explored key domains of cultural competence from the perspective of ethnically and linguistically diverse patients, concluding that TCAM and spirituality were among the factors influencing the quality of the medical encounter [76]. An open non-judgmental discussion, particularly in the oncology setting, would help overcome these prejudices, which are often unintentional and subconscious. This is especially important for patients with an “alternative” health belief model of care, which may contradict evidence-based research and clinical guidelines [77]. Such a discussion recognizes the fundamentals of medical ethics, respecting the patient’s autonomy, medical pluralism, and the mission to ensure accountability, based on perspectives related to beneficence and non-maleficence [78].

**Fig. 1:**
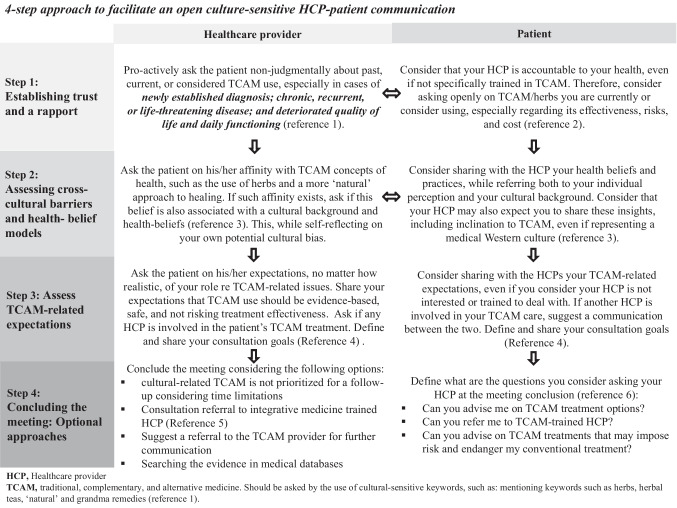
4-step approach to facilitate an open culture-sensitive HCP-patient communication [86–91]

Finally, including a translator or interpreter during the consultation may further advance cultural-sensitive communication, facilitating the partnership between HCPs and patients. A culturally-versed facilitator (for example, an Imam for Islamic patients treated in a non-Islamic setting) could also help the HCP in acquiring inter-cultural competencies. Table 2 present a list of suggested questions which can assist HCPs in facilitating a non-judgmental, culture-sensitive and open cross-cultural dialogue. Figure 1 presents a practical 4-step approach to facilitating HCP-patient communication, acknowledging perspectives from both sides and time-related restrictions of the clinic setting.

**Table 2:** Suggested questions to facilitate a non-judgmental, culture-sensitive and open cross-cultural dialogue

**Question goal**	**Questions to consider**
**Establishing an open non-judgmental setting for the TCAM discussion**	* Though I may (or may not) be) trained in alternative and complementary medicine, it is important for me to learn about your experience and beliefs regarding these approaches. Would you be willing to allow me to ask a number of open, non-judgmental questions?
**Initiating a discussion about TCAM use, including herbals, religious/spiritual practices; using terms associated with ‘alternative-natural’ medicine use**	* Are you presently taking or considering using any herbs or other traditional, ‘natural’, alternative, complementary (TCAM), religious or spiritual treatments for improving your health?
**Identifying the patient’s perspective on who should be included in the discussion on TCAM**	* Have you discussed the use of TCAM with your spouse, family member, TCAM practitioner, religious or spiritual practitioner? Have you thought about getting advice from other traditional healers, medical or non-medical professionals? Have you discussed this with a physician, nurse or other healthcare provider (e.g., pharmacist)?
**Identifying patient TCAM-related treatment goals**	* How do you perceive the goals of the treatment with TCAM? Is your primarily goal to relieve your symptoms and improve your quality of life? Or is it to “fight” or cure the disease, prolong life, “strengthen” your immune system, or another goal?
**Exploring the patient’s understanding regarding effectiveness, safety, quality, and cost of TCAM treatments**	* Are you aware of any benefits from the use of TCAM treatments? How about the potentially harmful effects of these modalities (toxic effects, negative herb–drug interactions)? Do you have any concerns about the quality of the herbal product, the professionalism of the TCAM practitioner, or the cost of treatment? Would you like my advice on these issues?
**Considering referral to an integrative healthcare provider for consultation**	* Have you considered consulting with an integrative physician who is trained in TCAM and familiar with complementary medicine? Would you like me to refer you to consultation with an integrative medicine practitioner?
**Sharing an opportunity for shared-reflection and ‘self-place’ (as a relaxation metaphor) for discussion**	*What are your expectations from me, as your HCP? How would you like me to be of further assistance? Do you feel that I am able/unable to answer your questions about the use of herbal medicine? Would you like me to involve another HCP (e.g., nurse) or another person from your family and community to discuss TCAM use?
**Inviting the patient to co-design the goals, plan and implementation of the IM treatment program**	* Would you be willing to participate in co-designing the TCAM treatment goals and plan? If you anticipate difficulties in implementing the treatment plan, will you be willing to share these with me? Would you be willing to suggest tips on how to overcome any of the barriers to our interaction? Would you consider inviting another person (e.g., family or community member; religious figure; TCAM therapist) to help implement the conventional medical recommendations? How about how to safely help you adhere to the oncology treatment regimen?

*TCAM* Traditional, complementary, alternative medicine, *IM* Integrative medicine

## Conclusions

The present review explores challenges facing TCAM-related cross-cultural communication, with the goal of providing HCPs with practical tools to facilitate a discussion with their patients, including those from minoritized groups with an affinity to TCAM-based health-belief model. The review was conducted by an international, multi-disciplinary team of researchers, though the available research addressing the study question remains extremely limited. A number of important questions remain unanswered: What is the prevalence of TCAM use in varied clinical settings among immigrants and refugees in the US and other industrialized countries? What is the number of integrative/TCAM-trained HCPs currently providing consultations to these patient populations and which are the modalities being used? Should a cultural-sensitive communication approach be adopted generally, or culturally-tailored to patients affiliated with different health-beliefs, cultures, and religions? What are the clinical outcomes (e.g., risk reduction of negative interactions between TCAM and medical treatments; adherence to medical treatment) of adopting a cultural-sensitive approach? To what extent does the cultural-sensitive approach lead to specific and measurable outcomes, vs. non-specific (e.g., empathic communication)? The present review provides practical recommendations for facilitating TCAM-related cross-cultural communication. At the same time, it highlights the need for evidence-based clinical guidelines on these aspects, while considering themes of safety/risk, medical education and implementation/feasibility of integrative medicine models in real-world clinical practice. This, while creating a collaborative multi-disciplinary initiative to develop and implement integrative medicine models within mental health care.

## Data Availability

No datasets were generated or analysed during the current study.
