# Correction to: Cross-Cultural Patient Counseling and Communication in the Integrative Medicine Setting: Respecting the Patient’s Health Belief Model of Care

**DOI:** 10.1007/s11920-024-01518-z

**Published:** 2024-07-17

**Authors:** Eran Ben-Arye, Gabriel Lopez, Maryam Rassouli, Miriam Ortiz, Holger Cramer, Noah Samuels

**Affiliations:** 1 Rappaport Faculty of Medicine, Technion-Israel Institute of Technology, Haifa, Israel; 2 Integrative Oncology Program, The Oncology Service, Lin, Zebulun, and Carmel Medical Centers, Clalit Health Services, Haifa, Israel; 3 Department of Palliative, Rehabilitation and Integrative Medicine, University of Texas, MD Anderson Cancer Center, Houston, TX USA; 4 Cancer Research Center, Shahid Beheshti University of Medical Sciences, Teheran, Iran; 5 Charité - Universit?tsmedizin Berlin, Corporate Member of Freie Universit?t Berlin and Humboldt-Universit?t zu Berlin, Institute of Social Medicine, Epidemiology and Health Economics, Berlin, Germany; 6 Institute of General Practice and Interprofessional Care, University Hospital T?bingen, T?bingen, Germany; 7 Robert Bosch Center for Integrative Medicine and Health, Bosch Health Campus, Stuttgart, Germany; 8 Center for Integrative Complementary Medicine, Shaarei Zedek Medical Center, Faculty of Medicine, Hebrew University of Jerusalem, Jerusalem, Israel

**Correction to: Current Psychiatry Reports**

10.1007/s11920-024-01515-2

The original version of this article unfortunately contained mistake in the Author’s Contribution statement. The corrected statement is as follows.

The original version of this paper has been corrected.

